# Exploring the Characteristics and Competencies of Successful Osteopathic Medical Students on General Surgery Clerkship

**DOI:** 10.7759/cureus.87897

**Published:** 2025-07-14

**Authors:** Ryan D Muchard, Trevor J Donnelly, Cole S Arnold, Richard R Thacker, Sherry L Roach

**Affiliations:** 1 Clinical Sciences, Alabama College of Osteopathic Medicine, Dothan, USA

**Keywords:** clinical clerkship, general surgery, medical school education, osteopathic medical student, preceptor, rotation, surgery, surgery rotation, surgical clerkship, surgical skills

## Abstract

Introduction

With the transition of the Comprehensive Osteopathic Medical Licensing Examination (COMLEX) Level 1 and United States Medical Licensing Exam (USMLE) Step 1 to pass/fail, medical students look for additional ways to demonstrate preparedness for surgical residencies. With a heavier emphasis on clinical rotation performance, it is imperative that students have the best understanding of preceptor expectations prior to the clinical training transition. This study aims to assess preceptor perceptions of what knowledge, characteristics, skills, and resources are most valuable to ensure student preparedness for and success during general surgical rotation.

Methods

A 10-question survey on the Qualtrics XM (Qualtrics, North Sydney, Australia) survey web-based platform was sent to general surgery preceptors (Doctor of Medicine (MD) and Doctor of Osteopathic Medicine (DO)) within the faculty network at the Alabama College of Osteopathic Medicine (ACOM). Survey responses were collected in rank order, with lower mean or median values indicating greater value. Median, mean values, and standard deviations are reported.

Results

Survey responses were completed in their entirety by 37.8% (25) of the surveyed population. The majority of respondents (72%) reported training over 10 osteopathic and allopathic medical students during their careers. Survey responses were completed in their entirety by 37.87% (n = 25) of the surveyed population. The majority of respondents (71.40%) reported training over 10 osteopathic and allopathic medical students during their careers. Surgeons ranked characteristics and qualities, pre-clinical knowledge, and patient management as the most important competencies (medians: 1, 2, and 3, respectively), above surgical knowledge, OR knowledge, and suturing (medians: 4-5). Sub-analyses favored sterile technique, simple interrupted sutures, differential diagnosis, enthusiasm, and anatomic knowledge over their counterparts; 44% of respondents stated that their expectations of students on general surgical rotation differ based on their preferred specialty of interest.

Conclusion

These findings emphasize that general surgery preceptors prioritize interpersonal qualities, such as enthusiasm and a willingness to learn, over technical skills during early clinical experiences. This suggests that fostering a proactive attitude and adaptability significantly enhances student performance and evaluations during surgical rotations. The emphasis on foundational knowledge and patient management skills over procedural expertise highlights the importance of cognitive preparedness in these settings.

These results suggest that medical schools should guide students to prioritize interpersonal and foundational competencies. By aligning preparation strategies with preceptor expectations, students may enhance their readiness for surgical rotations and improve clerkship performance, which is increasingly critical in the post-Step 1 pass/fail era. Future research could investigate how these competencies influence residency selection and identify ways to standardize preceptor expectations across institutions.

## Introduction

As the pathway to a surgical career becomes more competitive and complex, osteopathic medical students find themselves navigating a landscape that demands more than just academic knowledge -they must now cultivate specific competencies, demonstrate resilience, and embody the intangible qualities that make a successful surgeon. Clinical performance, letters of recommendation, and clerkship evaluations are key factors in determining residency candidacy. Yet, for many students, the expectations of surgical preceptors remain elusive, often leading to uncertainty and missed opportunities during their clerkships [1-2]. This study seeks to bridge that knowledge gap by capturing the insights of surgical preceptors on the knowledge, skills, and personal qualities they believe are essential for student success in the operating room and beyond. By understanding these preceptor-valued attributes, students can better prepare themselves for clinical training, positioning themselves as strong candidates for surgical residencies and setting the stage for a successful transition into their chosen field.

The shift of American medical school board exams to pass/fail scoring for the Comprehensive Osteopathic Medical Licensing Examination (COMLEX) Level 1 and United States Medical Licensing Examination (USMLE) Step 1 marks a pivotal change in medical education, significantly impacting the metrics used by residency programs to assess applicants [3]. With Level 1 and Step 1 no longer providing a quantitative ranking, clerkship performance and subjective assessments, such as letters of recommendation, are becoming primary tools for evaluating candidate readiness [4]. This transition places greater emphasis on the ability of osteopathic medical students to excel in clinical environments, particularly in high-stakes rotations such as general surgery, where preceptors closely observe direct patient care, procedural skills, and professionalism. However, the specific competencies and personal qualities that surgical preceptors prioritize remain unclear to many students, potentially leading to mismatches between student efforts and preceptor expectations. Understanding these priorities could provide students with targeted guidance, helping them align their preparation with the attributes that preceptors value most [5]. This study aims to bridge the knowledge gap by investigating preceptor perspectives on the essential knowledge, skills, and characteristics that contribute to success in general surgery clerkships, thereby informing students and medical education programs on how to prepare candidates for a career in surgery.

Moreover, while medical students begin their clinical rotations with a foundational knowledge base sufficient to pass board examinations, they often lack a clear understanding of the specific attributes and competencies that surgical preceptors deem essential for success. Preceptors assess students not only on technical knowledge but also on characteristics like teamwork, adaptability, professionalism, and enthusiasm for learning [6]. These non-technical qualities, which significantly contribute to clinical performance, are seldom emphasized in traditional preclinical education and can only be learned through firsthand experience in the operating room. Consequently, students entering general surgery clerkships may feel disoriented or uncertain about how to meet preceptors' expectations, which could impact their evaluations and their potential to secure a surgical residency position [7]. This uncertainty is especially pronounced within surgery, where the fast-paced, high-stakes environment can intensify feelings of inadequacy or underperformance among students.

The recent shift to a pass/fail grading system for Level 1 and Step 1 has amplified the importance of clerkship grades and subjective evaluations in the residency selection process. Without the once-quantitative score, program directors increasingly rely on clinical performance evaluations to distinguish between applicants, placing heightened scrutiny on students' clerkship records as indicators of their readiness for the rigors of residency. This transition has led residency programs to place greater weight on clerkship grades, viewing them as more reliable indicators of clinical aptitude and suitability for advanced training [8]. As a result, the ability to excel in clerkships, especially in competitive fields like surgery, is now pivotal to a student’s residency prospects. This new landscape highlights the importance for medical students to develop a deeper understanding of the qualities that surgical preceptors value and to tailor their preparation accordingly, thereby enabling them to better navigate the clinical environment and optimize their performance in ways that align with preceptor expectations.

This article was previously presented as an abstract and poster at the 2023 American College of Osteopathic Surgeons Medical Student Section (ACOS-MSS) Annual Spring Conference on April 29, 2023.

## Materials and methods

A descriptive survey study based on a review of relevant literature was conducted to gather insights into the competencies valued by general surgery preceptors, targeting faculty preceptors within the Alabama College of Osteopathic Medicine network. After Institutional review Board (IRB) approval, a structured, 10-question Qualtrics XM (Qualtrics, North Sydney, Australia) web-based platform survey was distributed via email to 66 general surgery preceptors (Doctor of Medicine (MD) and Doctor of Osteopathic Medicine (DO)) to assess their perspectives on the knowledge, characteristics, skills, and resources most critical for medical student success during general surgery clerkships. This survey was specifically designed to identify the attributes and skills that preceptors associate with ‘top-performing’ osteopathic students, allowing for a detailed understanding of preceptor expectations.

**Figure 1:**
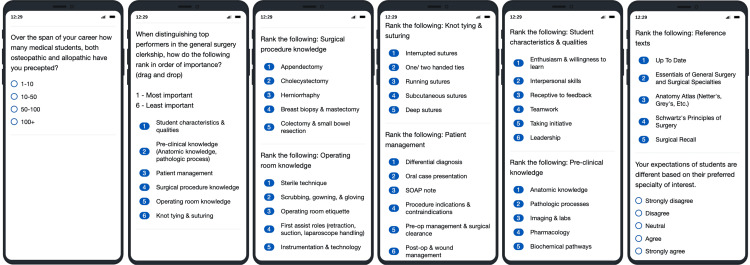
Survey questions Multiple choice (question 1). Ranking (questions 2-9): 1 - most important; 6 - least important. Likert scale (question 10).

The survey included multiple-choice, ranking, and Likert-scale questions. The second question asked preceptors to rank broad categories of student core competencies, including characteristics, procedural knowledge, clinical skills, and others, on a scale from one to six, with lower scores indicating higher perceived importance (Figure 1). The following seven questions were designed to break down each broad competency into subcategories for granular analysis; for example, "characteristics" were further analyzed based on enthusiasm, teamwork, and receptiveness to feedback, among other attributes. This approach provided an overarching view and detailed insights into the specific competencies preceptors prioritize in evaluating students.

Survey responses were collected over a one-month period, with three reminder emails sent two weeks apart to maximize response rates. Responses were collected anonymously to encourage honesty and were analyzed in terms of mean values and standard deviations to reflect the relative importance of each ranked competency and sub-competency. Mean scores for each item allowed for identifying competencies that preceptors consistently value, as well as those with higher variability. This ranking methodology ensured that the data accurately represented preceptor priorities, providing valuable, quantifiable insights into the characteristics defining high-achieving students in general surgery.

Data were analyzed using descriptive statistics, with an emphasis on identifying competencies ranked as high priority (i.e., those with lower mean or median values). This structured approach allows for a comprehensive understanding of preceptor expectations, offering actionable insights for students preparing for surgical rotations and providing potential guidelines for curriculum design.

## Results

**Figure 2:**
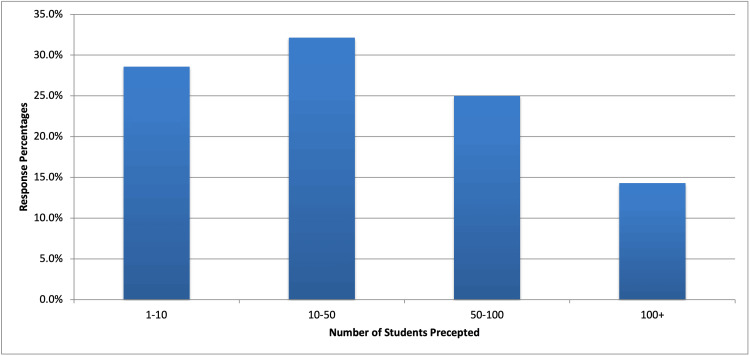
Number of medical students precepted Question 1: Over the span of your career, how many medical students, both osteopathic and allopathic, have you precepted? 1-10: 28.60%; 10-50: 32.10%; 50-100: 25.00%; 100+: 14.30%

Survey responses were completed in their entirety by 37.87% (n = 25) of the surveyed population. The majority of respondents (71.40%) reported training over ten osteopathic and allopathic medical students during their careers (Figure 2). Surgeons ranked core competencies, with one being the most important and six being the least important. Surgeons placed the greatest value on characteristics and qualities (median of 1), pre-clinical knowledge (2), and patient management (3), over surgical procedure knowledge (4), operating room knowledge (5), and knot tying and suturing (5) (Table 1). Further sub-analyses determined the ranked value of sterile technique (1) over instrumentation and technology (5), simple interrupted (2) over deep sutures (5), building a differential diagnosis (2) over pre-op management (4), enthusiasm and willingness to learn (1) over leadership qualities (6), and anatomic knowledge (1) over pharmacology knowledge (4) (Table 1).

**Table 1:** Survey results Ranking: Mean rank values with standard deviations and median rank values. 1 - most important; 6 - least important.

Rank Order Results	Mean ± SD	Median
Question 2		
Student characteristics and qualities	1.89 ± 1.29	1
Pre-clinical knowledge	2.00 ± 1.04	2
Patient management	2.79 ± 1.18	3
Surgical procedure knowledge	4.36 ± 1.14	4
Operating room knowledge	4.71 ± 0.92	5
Knot tying and suturing	5.25 ± 0.78	5
Question 3		
Appendectomy	1.96 ± 1.23	1
Cholecystectomy	2.26 ± 1.00	2
Herniorrhaphy	3.15 ± 1.15	3
Breast biopsy and mastectomy	3.59 ± 1.50	4
Colectomy and small bowel resection	4.04 ± 0.92	4
Question 4		
Sterile technique	1.50 ± 0.63	1
Scrubbing, gowning, and gloving	2.46 ± 0.91	3
Operating room etiquette	2.54 ± 1.18	2
First assist roles	4.04 ± 0.82	4
Instrumentation and technology	4.46 ± 0.87	5
Question 5		
Interrupted sutures	1.78 ± 0.83	2
One/two-handed ties	2.15 ± 1.27	2
Running sutures	3.07 ± 1.05	3
Subcutaneous sutures	3.33 ± 1.05	4
Deep sutures	4.67 ± 0.61	5
Question 6		
Differential diagnosis	2.21 ± 1.21	2
Oral case presentations	2.61 ± 1.37	2
Subjective, Objective, Assessment, and Plan (SOAP) note	3.43 ± 1.29	3
Procedure indications and contraindications	3.57 ± 1.29	4
Pre-op management and surgical clearance	4.04 ± 1.55	4
Post-op and wound management	5.14 ± 0.95	5
Question 7		
Enthusiasm	1.96 ± 1.35	1
Interpersonal skills	2.78 ± 1.34	2
Receptive to feedback	3.48 ± 1.29	3
Teamwork	3.56 ± 1.50	4
Taking initiative	3.81 ± 1.63	4
Leadership	5.41 ± 0.83	6
Question 8		
Anatomic knowledge	1.61 ± 0.98	1
Pathologic process	1.79 ± 0.62	2
Imaging and labs	3.11 ± 1.05	3
Pharmacology	4.04 ± 0.68	4
Biochemical pathways	4.46 ± 0.68	5
Question 9		
Essentials of General Surgery	2.58 ± 1.28	2
UpToDate	2.73 ± 1.35	2
Anatomy Atlas	2.96 ± 1.34	3
Schwartz’s Principles of Surgery	2.96 ± 1.43	3
Surgical Recall	3.77 ± 1.37	4

Ranked ANOVA statistical testing was run for the number of students precepted over the span of their career against the ranking core competencies, and no statistical significance was found between the number of students precepted and ranking order. However, a strong statistical significance was found between a higher number of students presented and the ranking of breast, biopsy, and mastectomy knowledge being lower (P = 0.0190). For preceptors of over 100 students, the average rank was 4.75 (with five being the lowest rank). No statistically significant relationships were found between the number of students taught and the other surgical procedure knowledge areas. Surgeons who taught 50 to 100 and 100+ ranked teamwork more important than groups with fewer students, with an average of 2.57 and 2.00, respectively. Demonstrating a statistically significant difference in ranking between the number of students precepted and the importance of teamwork (P = 0.00321). Ranked ANOVA, statistical testing, was run for the number of students precepted against all other subanalysis questions and answer choices, which yielded no other statistically significant results (Table 2).

**Table 2:** ANOVA table Dependent variables: Measured outcomes of interest (e.g., knowledge ranking or importance of teamwork). Group comparison: The specific groups analyzed based on the number of students precepted over their careers. Mean rank: Average rank values for the groups compared (if applicable). P-value: Probability value indicating statistical significance. Values less than 0.05 indicate significance. Statistical significance: Indicates whether the results showed a meaningful difference between groups.

Factor	Dependent Variable	Group Comparison	Mean Rank	P-value	Statistical Significance
Number of students precepted	Ranking of breast, biopsy, and mastectomy knowledge	Higher vs. lower number of students	4.75 vs. 3.02	0.019	Yes
Number of students precepted	Importance of teamwork	Higher vs. lower number of students	2.29 vs. 4.38	0.00321	Yes
Number of students precepted	All other subanalysis questions	Various	N/A	>0.05	No

**Figure 3:**
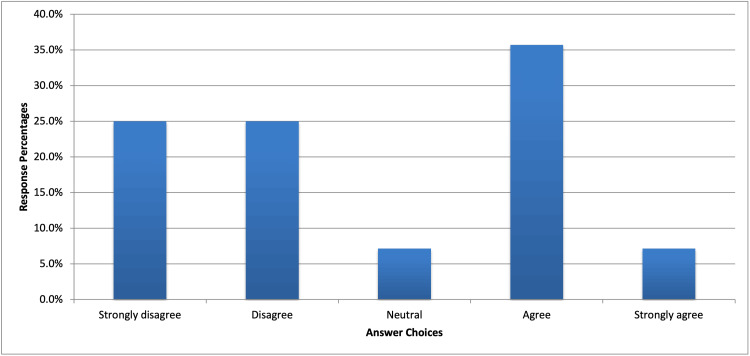
Preceptor expectations can differ based on the student's specialty of interest Question 10: Over the span of your career, how many medical students, both osteopathic and allopathic, have you precepted?

In question 10, 35.70% of respondents agreed, and 7.10% strongly agreed that their expectations of students on general surgical rotation differ based on their preferred specialty of interest. In comparison, 25% of preceptors disagreed, and 25% strongly disagreed; 7.10% were neutral on the matter (Figure 3). These findings provide valuable insights to medical students wishing to focus on competencies most valued by surgical preceptors.

## Discussion

The findings of this study highlight the core competencies and personal qualities that surgical preceptors prioritize for osteopathic medical student success during general surgery rotations. While the importance of nontechnical skills in surgical residency has been well documented, there is a relative lack of data specific to medical students. In one study, junior residents identified inherent personality traits and emotional intelligence as the most valuable qualities in their senior residents [9]. Similarly, clerkship preceptors consistently ranked student characteristics, such as enthusiasm and a willingness to learn, as highly important, followed closely by a strong foundational knowledge from preclinical training and the ability to manage patients effectively. These attributes were prioritized over technical skills, such as knot-tying, suturing, or procedural knowledge, suggesting that interpersonal qualities and cognitive preparedness are more critical for medical students in early clinical settings. While previous literature has emphasized surgery-specific preparation, such as cadaver dissections and skills laboratory experiences, these findings suggest that such technical training may be of lesser concern at this stage [10]. Demonstrating enthusiasm and a willingness to learn proved to be crucial for receiving an outstanding evaluation in general surgery clerkships. This emphasis may help guide students in their preparation, allowing them to focus on refining attitudes and intrinsic motivational traits to align with preceptor expectations.

Interestingly, the preference for enthusiasm and a proactive approach over leadership qualities further emphasizes the importance of a growth mindset. Preceptors appear to value students who are eager and open to learning more than those who may demonstrate overt leadership behaviors early in their rotations. This is in contrast to leadership as an important value to teach surgical residents during training [11]. This insight could influence medical schools’ preparation programs, perhaps promoting a focus on adaptability, active participation, and resilience in challenging environments, which may ultimately foster stronger performance and more favorable evaluations from preceptors. Medical school programs that emphasize resilience have been shown not only to be well-received by students but also to empower them as self-regulated learners [12]. This foundation could later serve as the basis for surgical residencies to instill leadership skills in their residents.

Additionally, the variation in expectations based on students' preferred specialties highlights an interesting area for further investigation. Nearly half of the respondents noted different expectations for students pursuing surgical versus non-surgical specialties, and there may be nuances in the competencies emphasized for each group. However, many students alter their specialty interests throughout medical school, particularly during their clinical years. If preceptors adjust their expectations based on a student's expressed interest, it could lead to variations in educational experiences, potentially influencing their final specialty choice in either encouraging them to pursue surgery or leading them to choose a different specialty [13] This insight begs further research as to how targeted preparation based on career interest could alter student success and alignment with preceptor expectations, particularly for those aiming to enter surgical residency programs.

The value of nontechnical skills in surgical education has been increasingly emphasized in recent literature, mirroring the findings of this study. While technical competence remains a cornerstone of surgical training, qualities such as communication, adaptability, and teamwork have gained recognition as essential for both educational development and patient safety [14-17]. These competencies are no longer viewed as supplementary but rather as integral components of a well-rounded surgical education. This broader perspective aligns with our findings, which indicate that interpersonal qualities were rated more highly than procedural abilities in the context of medical student performance. The emphasis on these traits during undergraduate medical education may reflect a growing awareness that successful surgical trainees must first demonstrate cognitive readiness and emotional intelligence before developing more advanced technical capabilities.

Similarly, student engagement and proactive attitudes have emerged as key drivers of meaningful learning experiences during surgical clerkships. Recent studies have identified eagerness, humility, confidence, and teamwork as qualities that enhance teaching interactions and foster stronger student-preceptor relationships [18-20]. These findings resonate with our data, in which enthusiasm and willingness to learn consistently ranked above leadership or procedural knowledge. A favorable learning climate, characterized by timely feedback, inclusion, and support, has also been shown to contribute to more effective student development. Together, these insights support the idea that early surgical education should prioritize emotional intelligence, receptiveness, and interpersonal engagement, qualities that may be more predictive of long-term success than early procedural expertise.

In response to this evolving educational landscape, surgical educator frameworks now include competencies such as psychological safety, learner-centeredness, and adaptability to individual student needs. These elements have become central to competency-based medical education, with tools such as Entrustable Professional Activities (EPAs) emerging to ensure that trainees progress based on skill demonstration rather than time alone [21,22]. This shift echoes the need for a more holistic assessment strategy in undergraduate surgical rotations, where student growth is not solely defined by technical proficiency. Notably, our findings suggest that enthusiasm and diagnostic reasoning, two early, teachable attributes, may better align with these evolving models than procedural repetition alone.

Additionally, there is growing awareness of how personality characteristics influence trainee selection and performance. Evaluators have increasingly placed higher importance on interpersonal effectiveness and communication skills than on raw technical ability [23,24]. This is particularly relevant for international medical graduates and students from diverse educational backgrounds, where perceived differences in essential competencies may exist. Bridging these gaps through structured feedback, mentorship, and faculty development initiatives could help standardize expectations and promote equity in surgical education. These observations further reinforce the notion that early clinical success is grounded more in attitude and adaptability than in technical precision.

Finally, while collaboration, leadership, and management are increasingly cited as core surgical competencies, curricular mapping studies reveal that their integration into undergraduate education remains limited [15-17,25]. Simulation-based training and structured assessment tools such as the Non-Technical Skills for Surgeons (NOTSS) framework offer promising approaches for teaching and evaluating these competencies. As medical schools and clerkship directors continue to refine curricula in the post-Step 1 era, an emphasis on structured non-technical skill development, supported by validated tools and standardized expectations, may better prepare students for both residency selection and the collaborative demands of surgical practice.

Limitations

This study has several limitations that should be noted. First, the survey sample size was relatively small, with only 25 completed responses, which may limit the generalizability of the findings to broader populations of surgical preceptors. Second, the study was conducted within a single osteopathic medical college faculty network, potentially introducing regional or institutional bias. Third, the survey’s rank-order design may have led to variability in how respondents interpreted the importance of each competency. Fourth, multiple statistical comparisons were conducted without formal correction, as the study was exploratory in nature; thus, significant p-values should be interpreted with caution due to the increased risk of Type I error. Finally, while the survey results provide valuable insights, further studies with larger, multi-institutional cohorts and qualitative methodologies may be needed to confirm these findings and explore additional perspectives.

## Conclusions

This study provides critical insights into the competencies and characteristics valued by surgical preceptors in general surgery rotations, emphasizing the importance of interpersonal qualities, foundational knowledge, and patient management skills over technical skills like suturing and procedural knowledge. Enthusiasm, willingness to learn, and adaptability were highly valued qualities, suggesting that preceptors might prioritize a growth mindset and proactive student engagement. Additionally, the potential variation in expectations based on a student's preferred specialty underscores the need for targeted preparation for those aiming for surgical careers. These findings can guide medical students and educational programs to focus on competencies that align with preceptor expectations, ultimately improving student performance and preparedness for surgical residency. Future studies should consider expanding the scope of this research to validate findings across a broader range of institutions and specialties.
